# Clinical, biochemical and molecular spectrum of acute neuronopathic type 2 Gaucher disease from India

**DOI:** 10.1186/s12887-026-07232-4

**Published:** 2026-06-27

**Authors:** Jayesh Sheth, Aadhira Nair, Riddhi Bhavsar, Mamta Muranjan, Sheela Nampoothiri, Dhanya Yesodharan, Anupriya Kaur, Raju C Shah, Prajnya Ranganath, Shagun Agrawal, Aditi Baluni, Niyati Saini, Ashwin Dalal, Frenny Sheth, Harsh Sheth

**Affiliations:** 1 FRIGE’s Institute of Human Genetics, FRIGE House, Satellite, Ahmedabad, India; 2 KEM Hospital, Mumbai, India; 3 Department of Pediatrics, Amrita School of Medicine, Kochi, India; 4 PGIMER, Chandigarh, India; 5 Ankur Institute of Child Health, Ahmedabad, India; 6 Department of Medical Genetics, NIMS, Hyderabad, India; 7 BRIC-Centre for DNA Fingerprinting and Diagnostics, Hyderabad, India

**Keywords:** Type 2 Gaucher disease, GBA1, neuronopathic, P.Leu483Pro, P.Met124Arg, Recurrent variant, Oculomotor apraxia, And dysphagia

## Abstract

**Background:**

Gaucher disease (GD) is one of the most common lysosomal storage disorders, and has three clinical subtypes (Type 1, 2 and 3). Of the three subtypes, type 2 GD (GD2), also referred to as acute infantile neuronopathic GD is the most severe type. It is characterized by early and profound central nervous system involvement, and patients succumb to disease before two years of age due to severe neurological deterioration and associated complications. It is the rarest of the three subtypes with a reported proportion of < 1% among all Gaucher subtypes. Furthermore, data from the Indian subcontinent on clinical profiles, molecular spectrum, and outcomes in GD2 remain scarce. The present study describes the clinical, biochemical, and molecular spectrum of GD2 cases in an Indian cohort.

**Methods:**

This is a retrospective study comprising 19 patients referred by pediatricians from across India, who were diagnosed with GD based on β-Glucosidase enzyme activity and *GBA* gene study. Plasma chitotriosidase activity and β-glucosidase activity were measured in plasma sample and leukocytes respectively using fluorometric enzyme assays. Molecular confirmation was performed in all 19 patients by using one of the following tests: polymerase chain reaction-restriction fragment length polymorphism (PCR-RFLP), Sanger sequencing, targeted panel or whole exome sequencing study.

**Results:**

The common clinical signs observed in the study cohort were hepatosplenomegaly, thrombocytopenia, oculomotor signs, failure to thrive and feeding difficulties. Molecular study revealed causative variants distributed across exons 3, 4, 5, 6, 7, 9, 10, and 11 of the *GBA1* gene, with exon 10 being the most common (*n* = 11/19). The variant c.1448T > C (p.Leu483Pro) was identified in a compound heterozygous state in majority cases (*n* = 10/19). Furthermore, we also identified a rare recurrent missense variant c.371T > G (p.Met124Arg) in exon 4 of the *GBA1* gene in 4 patients with GD2 from Gujarat, suggesting the possibility of a founder effect. Computational protein modeling and in-silico analysis predicted a destabilizing effect on GBA1 protein structure and stability.

**Conclusion:**

Overall, the present study represents the largest case series of type 2 Gaucher disease reported from India to date and provides important insights into its clinical and molecular spectrum in the Indian population.

**Supplementary Information:**

The online version contains supplementary material available at https://doi.org/10.1186/s12887-026-07232-4.

## Introduction

Gaucher disease (GD) is the most common lysosomal storage disorder, caused by biallelic pathogenic variants in the *GBA1* gene [1]. It encodes the lysosomal enzyme glucocerebrosidase, deficiency of which leads to accumulation of glucocerebroside within macrophages, eventually affecting multiple systems including the hematopoietic, visceral, skeletal, and neurological systems [2, 3]. There are three clinical subtypes of GD based on the clinical signs, onset age and involvement of the central nervous system [4]. Type 1 GD is chronic, purely visceral, and mainly characterized by an enlargement of spleen and liver, thrombocytopenia, anemia, and skeletal involvement with no neurological involvement. Subacute neuronopathic or type 3 GD (GD3) is a heterogeneous subtype encompassing a variable degree of visceral involvement combined with diverse neurological and cardiac involvement. Acute neuronopathic or type 2 GD (GD2) is characterized by an early and rapid neurological degeneration, with prominent brainstem involvement associated with splenomegaly, pulmonary and hematological signs. The clinical signs are evident in the first months of life, resulting in progressive neurodegeneration and mortality.

Type 2 Gaucher disease (GD2), also referred to as acute infantile neuronopathic Gaucher disease, was first described by Oberling and Woringer in 1927 [5]. Its estimated frequency is 1 in 150,000 [6] and it constitutes < 1% of all the Gaucher types [2]. Affected infants commonly present with hepatosplenomegaly, dysphagia, failure to thrive and skin manifestations [7, 8]. Primary CNS findings include progressive neurologic deterioration, poor suck and swallow reflexes, hypotonia, convulsions, microcephaly, apathy, hypokinesia or akinesia, opisthotonus, and enlarged cerebral ventricles [3]. Most patients with GD2 succumb to disease before two years of age due to severe neurological deterioration and associated feeding and respiratory complications [9].

Type 2 Gaucher disease (GD2) is rare compared to the other two subtypes, however this likely reflects the short lives of these infants, in the most severe forms, and the diagnosis is often not established before death. Although GD2 constitutes a small proportion of the total Gaucher cases, recent case series have highlighted a broader phenotypic spectrum than previously recognized [10]. These patients deteriorate rapidly but variably, typically over a period of up to two years, with bulbar symptoms necessitating airway interventions (e.g. tracheostomy) and loss of swallowing function. Many infants develop motor and tone problems, and some evolve to develop seizures. The original case of GD2 reported by Oberling described infants presenting at 5 months of age, or earlier, with significant growth failure, loss of visual attentiveness, strabismus, opisthotonus, increased tone, loss of ability to feed and death before 12 months with laryngospasm and asphyxia, and in some, myoclonus [5].

Even though early descriptions emphasized uniform clinical presentations, evolving evidence from larger cohorts has demonstrated considerable heterogeneity in age at onset, severity, and constellation of visceral and neurological features [9, 11]. This phenotypic diversity complicates both clinical classification and prognostication. The largest recent systematic analysis of GD2 involving a series of internationally referred patients combined with historical cases totaling 250 individuals underscored the wide clinical and genotypic variability within patients classified under the single “Type 2” label [10]. They proposed a clinical categorization based on age at presentation and predominant clinical features to better stratify outcomes and support genotype-phenotype analyses. Likewise, recent small single-centre cohort studies and case reports have also contributed to the understanding of GD2 by identifying novel *GBA1* mutations and detailing unique clinical presentations [12–14].

Despite these insights, there remains a scarcity of data from the Indian subcontinent on clinical profiles, molecular spectrum, and outcomes in GD2. Given the importance of population-specific genetic backgrounds and potential modifier effects on Gaucher disease progression, detailed case series from diverse ethnic and geographic settings are crucial. The present study aims to describe the clinical, biochemical, and molecular spectrum of GD2 cases from a tertiary centre in India, to broaden understanding of how this neuronopathic form manifests in this population. This study also describes a rare recurrent missense variant in the *GBA1* gene identified in patients from the western part of India, predominantly Gujarat. Overall, the present data will contribute to global efforts in refining GD2 classification and management strategies.

## Methods

### Patient cohort details

The present study comprised − 19 patients referred by pediatricians from across India, includeing 6 patients from Gujarat, 5 from Maharashtra, 2 each from Karnataka, Chandigarh and Kerala, and one case each from New Delhi and Telangana. These cases were pooled over a 10-year period (2016–2025). Among postnatal cases, the age at presentation ranged from 19 days to 1.1 years; two cases were identified prenatally. Informed consent for the investigations was obtained from parents of all patients. The Institutional ethics committee at FRIGE’s Institute of Human Genetics approved this study in accordance with the Helsinki declaration.

### Clinical categorization of patients

Based on the clinical characteristics, age at symptom onset, age at presentation, age at death, rate of neurological progression, and family history, all 19 patients were classified according to the five clinical categories proposed by Donald et al. 2025 [10]. These categories were used to distinguish severe neuronopathic Gaucher disease type 2 (GD2) from the more chronic neuronopathic phenotype of GD3. The five categories include: (i) Gaucher-related hydrops, characterized by fetal or neonatal presentation with hydrops fetalis, polyhydramnios, hepatosplenomegaly, collodion skin, and severe birth compromise; (ii) neonatal inflammatory GD, presenting within the first month of life with severe visceral involvement, thrombocytopenia, elevated inflammatory markers, and frequently associated ichthyosis or collodion skin; (iii) neonatal neurodegenerative GD, presenting in the neonatal period with rapidly progressive neurological manifestations such as bulbar dysfunction, opisthotonus, dystonia, and feeding difficulties; (iv) early infantile neurodegenerative GD, characterized by symptom onset between 1 and 6 months of age with prominent neurological involvement including bulbar dysfunction, apnea, movement disorders, seizures, and rapid neurodegeneration; and (v) late infantile neurodegenerative GD, presenting between 6 and 12 months of age with visceral disease accompanied by progressive neurological manifestations.

### Biochemical investigations

A peripheral blood sample (3-4 ml) collected in an EDTA vial from all patients was subjected to plasma separation. For performing biochemical analysis, leukocytes were separated from whole blood as described previously [15] and stored at -20 °C until further testing. For prenatal samples, enzyme study was performed using cultured amniotic cells.

### Plasma chitotriosidase testing

The chitotriosidase activity was measured in blood plasma using a previously described protocol [16]. In brief, the plasma mixed with 4-MU (4-methylumbelliferryl b-D-N, N,N′,N″- triacetylchitotrioside) substrate was incubated at 37 °C for 15 min. Fluorescence was measured using a photo-fluorometer with 360 nm excitation (primary) and 465 nm emission (secondary).

### Enzyme assay

The substrate 4-methylumbelliferyl-β-D-glucopyranoside was used to measure the Lysosomal hydrolase enzyme (β-glucosidase) activity from leukocytes using fluorometric assay [17].

### DNA extraction and storage

Genomic DNA was extracted from the peripheral blood of samples using the salting out technique [18]. For prenatal samples, genomic DNA was extracted from amniotic fluid sample. The genomic DNA was quantified using a QIAxpert (Cat. No: 9002340) from Qiagen, run on 2% agarose gel to check its quality and was then stored at -20℃ until further use.

### Preliminary testing of the p.Leu483Pro variant in the *GBA1* gene

The DNA samples of all 19 patients were amplified using primers mentioned in the Additional file 1 to detect the common variant p.Leu483Pro in the *GBA1* gene. Amplification conditions included an initial denaturation at 94℃ for 5 min, followed by 30 cycles each consisting of 1 min denaturation at 94℃ for 45 s of annealing at temperature 61℃, and 45 s extension at 72℃. The PCR product was then subjected to restriction enzyme digestion using MspI (New England Biolabs). For this, 10 µL of PCR product was incubated with 0.5 µL of MspI enzyme (10 U/mL) at 37℃ for 3 h. The digested product was then separated on a 2.5% agarose gel. On the basis of the size of different bands obtained, the variant p.Arg502Cys and p.Leu483Pro can be identified. The variant p.Arg502Cys was not detected in any of the 19 patients in the present study.

### Sanger sequencing

Genomic DNA extracted from 19 patients was subjected to PCR for amplification of the *GBA1* gene containing 11 exons. Gene-specific primer sets targeting pseudogene divergent regions were used for amplification of the functional gene only, as described previously [19] (see Additional file 1). The cycling conditions were as follows: initial denaturation (94 °C; 5 min), followed by 35 cycles of denaturation (94 °C; 30 s), annealing (61 °C; 45 s), elongation (72 °C; 45 s), and final elongation (72 °C; 10 min). Following this, the PCR products were purified using Exo-SAP-IT™ (USB Corporation, USA) and subjected to di-deoxy chain termination protocol using BigDye Terminator v3.1 cycle sequencing kit (Thermo Fisher Scientific, USA). Capillary electrophoresis was performed using an automated sequencer SeqStudio (Applied Biosystem, USA). Sequences were assessed by comparing with the hg19/GRCh37 genomic reference sequence of the *GBA1* gene using NCBI-BLAST (https://blast.ncbi.nlm.nih.gov/Blast.cgi). Variant nomenclature follows the Human Genome Variation Society (HGVS) guidelines using the precursor protein numbering, which includes the 39-amino acid leader sequence (e.g., p.Met124Arg). Traditional numbering, which excludes the leader sequence and refers to the mature protein, is used in figures for structural context (e.g., p.Met124Arg as M85R).

### Targeted single molecule molecular inversion probe (smMIP)-next generation sequencing (NGS) assay and Whole-Exome Sequencing (WES)

Targeted sequencing using a smMIP-NGS assay was performed on genomic DNA extracted from one patient, based on strong clinical suspicion of Gaucher disease. Briefly, 100 ng of genomic DNA was used for targeted capture of genomic regions encompassing 23 genes associated with 29 lysosomal storage disorders (LSD), as previously described [20]. Captured PCR products were pooled in equimolar proportions, purified using AMPure XP beads, and sequenced on the Illumina MiSeq platform (Illumina, USA) using custom sequencing primers with 2 × 156 bp paired-end reads.

Whole-exome sequencing (WES) was performed for one patient using the Twist Human Core Exome kit (Twist Biosciences, USA). Libraries were sequenced on the Illumina NovaSeq 6000 platform (Illumina, USA) with paired-end reads and a mean target coverage of ~ 100x.

### Bioinformatics and variant analysis

FASTQ files from both smMIP-NGS and WES were aligned to the GRCh37/hg19 reference genome using BWA-MEM v0.7.12 [21]. Variant calling for SNVs and indels was performed using GATK HaplotypeCaller v4.1.2, following base quality recalibration and GATK best-practice workflows [22]. Copy number variants (CNVs), including single-exon events in smMIP-NGS data, were detected using DECoN v1.0.1 [23] with batch normalization using ≥ 17 samples. CNVs from WES data were identified using CNVRobot v4.0.0 (https://github.com/AnetaMikulasova/CNVRobot).

Variant annotation and prioritization were performed using Exomiser v12 [24], incorporating HPO-based phenotype matching and multiple in silico prediction tools (SIFT, PolyPhen-2, MutationTaster, CADD, REVEL) and databases (dbSNP, gnomAD, ClinVar). Variants with MAF > 1% were excluded using 1000 Genomes, TopMed, and gnomAD. Filtering retained non-synonymous and canonical splice-site variants with read depth > 20x. Candidate variants were interpreted according to ACMG-AMP and ClinGen guidelines [25, 26], integrating population frequency, evolutionary conservation, computational predictions, and available functional data.

### In-silico analysis

The prediction of missense variants was done by using various pathogenicity prediction tools including Mutation Taster (http://www.mutationtaster.org), Mutation Assessor (http://www.mutationassessor.org), SIFT/PROVEAN (http://provean.jcvi.org) and PolyPhen-2 (http://genetics.bwh.harvard.edu/pph2). Additionally, functional impact and pathogenicity scores were obtained using MutPred2 (https://mutpred.mutdb.org/), a probabilistic tool that models structural and mechanistic signatures of amino acid changes.

### Protein visualization and structure analysis

The protein sequence of GBA1 in FASTA format and variant information was retrieved from UniProt database with UniProtKB ID-P04062. The three-dimensional (3D) structure of the GBA1 protein with the resolution of 2.5 Å was obtained from the Protein Data Bank with ID 3GXD. This structure was selected because it represents the human GBA1 protein, provides extensive coverage of the mature enzyme, and contains the variant residue Met124 within a well-resolved region of the protein. The structure, determined by X-ray crystallography at 2.5 Å resolution, enables reliable assessment of residue-level interactions and has been widely utilized in previous structural studies of GBA1 variants. The amino acid numbering of the sequence and structure were correlated using Multiple Sequence Alignment (MSA) using Clustal Omega [27]. The mutations were manually added in the native protein sequence (PDB ID: 3GXD).

Three-dimensional structures of wildtype and mutant GBA1 proteins were visualized using PyMOL. The Mutagenesis Wizard function was used to introduce specific amino acid changes. The impact of the missense variant p.Met124Arg on protein stability was assessed using iStable [28] and DynaMut2 [29] that estimate changes in thermodynamic stability (ΔΔG) upon mutation. Atomic-level interaction profiling was performed using Arpeggio [30], which computes and classifies interatomic interactions including hydrogen bonds, hydrophobic interactions, ionic interactions, π-stacking, and van der Waals contacts.

## Results

### Study cohort

The present study describes 19 patients diagnosed with type 2 Gaucher disease using lysosomal enzyme study and/or molecular study as shown in (Table 1). There were 9 females (47.4%),8 males (42.1%), and two prenatal cases (10.5%). Consanguinity was documented in one family, while the remaining families reported no consanguinity. A positive family history suggestive of a similar disorder or early sibling death was noted in 3 patients.

**Table 1:** Clinical signs, enzyme activity and variant details for all the 19 patients with type 2 Gaucher disease included in the present study

Patient ID	Age (Age at symptom onset)	Region	Gender	Consanguinity/ Family history	TC	HSM	OS	Regression	FD; FTT	SZ	SP	Hypotonia/Hypertonia	BME	Others	Plasma chitotriosidase (nmol/h/ml)/ β-glucosidase enzyme activity (nmol/h/mg protein)	Molecular study (Zygosity)	ACMG classification	Status (age at death/ follow-up details)	Clinical category ^e^
P1	1.1 Y (Not available)	Maharashtra	F	Yes^a, b^	-	✓	✓	✓	✓	-	-	-	Not done	None	16390.8 / 0.31	c.1448T > C p.Leu483Pro Exon 10 (Homo)	P	Deceased (lost to follow-up)	Possibly Late-IN
P2	9 months (Not available)	Maharashtra	M	No	-	✓	✓	✓	✓	-	-	-	Not done	None	54916.9 / 0.54	c.721G > A p.Gly241Arg Exon 6 (Homo)	P	Deceased (lost to follow-up)	Possibly Late-IN
P3	19 days (Since birth)	Gujarat	M	No	✓	✓	-	-	✓	-	-	-	Not done	Pulmonary infection	1123.6 / Not done	c.472 A > G p.Ile158Val Exon 5/ RecNcil (Comp het)	LP/ P	Deceased (lost to follow-up)	NI
P4	8 months (Not available)	Chandigarh	F	No/ Yes^c^	-	✓	✓	✓	✓	-	-	✓(Hypertonia)	Not done	None	21748.1 / 1.0	c.1448T > C p.Leu483Pro Exon 10/ RecNcil (Comp het)	P	Deceased (lost to follow-up)	Possibly Late-IN
P5	10 months (Not available)	Chandigarh	M	No	-	✓	✓	✓	✓	-	-	✓ (Hypotonia)	Gaucher cells	Convergent squint	34044.5 / 0.62	c.1448T > C p.Leu483Pro/ c.1448T > G p.Leu483 Arg Exon 10 (Comp het)	P	Deceased (lost to follow-up)	Possibly Late-IN
P6	1 Y (9 months)	Gujarat	F	No	✓	✓	-	✓	✓	-	-	✓ (Hypotonia)	Gaucher cells	No neck holding, pallor, mild icterus	22,977 / 0.4	c.260G > A p.Arg87Gln Exon 3 (Homo)	P	Deceased (1.3 years)	Late-IN
P7	1 Y (Before 6 months)	Karnataka	M	No	-	✓	-	✓	✓	-	-	✓ (Hypotonia)	Gaucher cells	None	Not done / 1.07	c.1448T > C p.Leu483Pro Exon 10/ c.475 C > T p.Arg159Trp Exon 5 (Comp het)	P	Deceased (1.8 years)	Early-IN
P8	9 months (Not available)	Delhi	F	No	-	-	-	-	✓	✓	✓	✓ (Hypotonia)	Not done	Developmental delay	11450.27 / 1.3	c.1448T > C p.Leu483Pro Exon 10/ c.242G > A p.Ser81Asn Exon 3 (Comp het)	P/ LP	Deceased (10 months)	Possibly Early-IN
P9	6 months (5 months)	Gujarat	F	No	-	✓	-	-	✓	✓	-	✓ (Hypotonia)	Not done	Recurrent upper respiratory infections, microcephaly, micrognathia, fever-associated seizures	11,776 / 0.23	c.1448T > C p.Leu483Pro Exon 10/ c.371T > G p.Met124Arg Exon 4 (Comp het)	P/ LP	Deceased (8 months)	Early-IN
P10	6 months (2.5 months)	Gujarat	M	No	-	✓	✓	-	✓	-	-	-	Not done	Dystonia	26,100 / 2.6	c.1448T > C p.Leu483Pro Exon 10/ c.371T > G p.Met124Arg Exon 4 (Comp het)	P/ LP	Deceased (lost to follow-up)	Early-IN
P11	8 months (3 months)	Maharashtra	M	No	-	✓	-	✓	✓		✓	✓ (Hypotonia)	Not done	Mild pallor, hospitalized due to acute choking episode with gasping respirations, hypo pigmented lesions on the trunk	0 / 0.81	c.1448T > C p.Leu483Pro Exon 10/ c.1255G > C p.Asp419His Exon 9 (Comp het)	P/ LP	Deceased (lost to follow-up)	Early-IN
P12	9 months (5 months)	Gujarat	M	No	✓	✓	✓	✓	✓	-	-	✓ (Hypotonia)	Not done	Recurrent lung infections, brain MRI- mild delay in myelination	16321.8 / 0.7	c.1448T > C p.Leu483Pro Exon 10/ c.371T > G p.Met124Arg Exon 4 (Comp het)	P/ LP	Deceased (lost to follow-up)	Early-IN
P13	6 months (3.5 months)	Gujarat	F	No	✓	✓	✓	-	✓	-	-	✓ (Hypotonia)	Not done	Developmental delay, poor weight gain, Erlenmeyer flask deformity of the distal femoral metaphysis on X-ray of the upper and lower limbs	23116.4 / 0.3	c.1448T > C p.Leu483Pro Exon 10/ c.371T > C p.Met124Arg Exon 4 (Comp het)	P/ LP	Alive (was no ERT for four months, discontinued since last 2 months, no improvement)	Early-IN
P14	*In-utero* (Not available)	Telangana	UK	No	-	-	-	-	-	-	-	-	Not applicable	Septated cystic hygroma, nonimmune fetal hydrops	Not done / ^#^7.15	Complex C (Homo)	P	Abortion at 17 weeks	Hydrops
P15	*In-utero* (21 weeks)	Karnataka	UK	No	-	-	-	-	-	-	-	-	Not applicable	Skeletal dysplasia, short long bones, hydrops, polydactyly	Not done/ ^#^0.0	RecNcil (Hetero)	P	Abortion (lost to follow-up)	Hydrops
P16	11 months (Not available)	Kerala	F	Yes^d^	-	✓	✓	✓	✓	✓	✓	✓(Hypertonia)	Gaucher cells	Right convergent squint, aspiration of feeds, restricted upward gaze, nasal regurgitation	Not done/ 0.63	c.866G > C p.Gly289Ala Exon 7 (Homo)	P	Abortion (13 months)	Late-IN
P17	1 Y (Not available)	Kerala	M	No	✓	✓	✓	-	✓	-	✓	Facial dystonia, opisthotonic posturing	Gaucher cells	Anemia by 8 months, significant stridor, facial grimace, alternate convergent squint, oculomotor apraxia	21,915/ 1.3	c.508 C > T p. Arg170Cys Exon 5/ c.1448T > C p.Leu483Pro Exon 10 (Comp het)	P	Deceased (15 months)	Late-IN
P18	1 Y (7 months)	Maharashtra	F	No	✓	✓	✓	✓	✓	-	-	-	Gaucher cells	Eyes- disc pallor indicating optic atrophy, early macular deposits at fovea surrounded by grayish zones suggestive of early cherry red spot	0.0/ 1.8	RecNcil+55 bp deletion/ c.1603 C > T p.Arg535Cys Exon 11 (Comp het)	P	Deceased (13 months)	Late-IN
P19	1.6 Y (5 months)	Maharashtra	F	No	✓	✓	✓	✓	-; ✓	✓	✓	-	Gaucher cells	Microcephaly, DTR 3 + with ankle clonus, extensor plantar response	Not done	Complex C/ c.680 A > G p.Asp227Ser Exon 6 (Comp het)	P	Deceased (2 years)	Early-IN

Abbreviations: *M *Male, *F *Female, *UK *Unknown, *TC *Thrombocytopenia, *HSM *Hepatosplenomegaly, *OS *Oculomotor signs, *FD *Feeding difficulties, *FTT *Failure to thrive, *SZ *Seizures, *SP *Spasticity, *BME *Bone marrow examination findings, *Homo *homozygous, *Comp het* compound heterozygous, *Hetero *heterozygous,* P *Pathogenic, *LP *Likely Pathogenic

^(#)^ Sample type was: cultured amniotic fluid cells (Reference range: 320–500 nmol/h/mg protein), Reference range for leukocyte sample: 4.0–32.0 nmol/h/mg protein

^a^ Third degree, ^b^ Previous elder sibling (male) died at 2.5 years, ^c^ Previous elder sibling (female) died at 1 year of age, ^d^ 4th degree once removed, ^e^ Clinical categorization done as per Donald et al. 2025, Early-IN: Early infantile neurodegenerative, Late-IN: Late infantile neurodegenerative, NI- Neonatal inflammatory, Hydrops- Gaucher related hydrops

### Clinical details

**Fig. 1:**
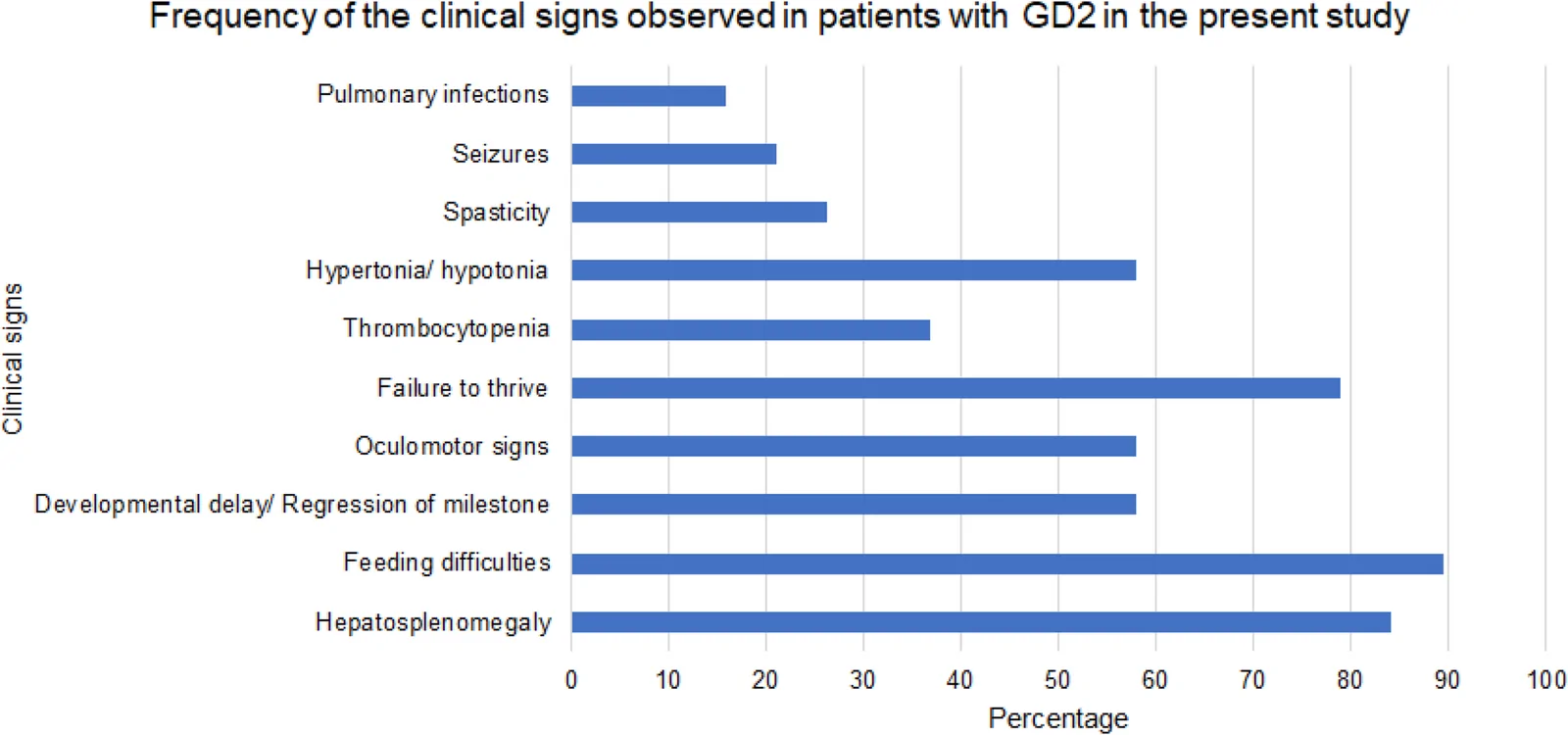
Frequency of the clinical signs observed in patients with GD2 in the present study

The key clinical signs noted among patients in the present study included hepatosplenomegaly, thrombocytopenia, anemia, developmental delay, oculomotor apraxia and regression of milestones. Feeding difficulties, including vomiting, and difficulty in swallowing food, were common presenting signs. Failure to thrive was a common clinical feature in these patients. Additionally, neurological manifestations such as seizures were observed in four patients and spasticity was observed in five patients. Oculomotor signs were observed in nine patients (Fig. 1). Bone marrow examination in seven patients revealed presence of Gaucher cells. Table 1 describes the key clinical signs, enzyme activity and variant details for all the 19 patients included in the study. In both prenatal cases, the presenting feature was nonimmune fetal hydrops. Based on the classification system proposed by Donald et al. 2025 [10], the clinical phenotypes in the present cohort were categorized as follows: 2 patients with Gaucher-related hydrops, 1 patient with neonatal inflammatory GD, 7 patients with early-infantile neurodegenerative GD, and 4 patients with late-infantile neurodegenerative GD. In addition, 4 patients were classified as possibly late-infantile neurodegenerative GD and 1 patient as possibly early-infantile neurodegenerative GD due to limited longitudinal follow-up data and absence of precise age at symptom onset. On follow-up of all 19 patients, eighteen died during the course of the disease, consistent with the well-established natural history of GD2. However, the age at death was available only for a subset of patients and is recorded in Table 1 where known. One patient is alive at the time of reporting and received enzyme replacement therapy (ERT) for approximately four months, following which it was discontinued due to the absence of clinical improvement.

### Biochemical analysis

Plasma chitotriosidase levels were available for 14 patients and were markedly elevated except for two patients (P11 and P18). In both these patients, plasma chitotriosidase activity was undetectable, which may reflect *CHIT1* gene homozygous duplication rather than disease biomarker levels. The mean chitotriosidase level was 21823.36 ± 13357.51 nmol/h/mL plasma (range: 0–54,916.9 nmol/h/mL). β-glucosidase enzyme activity in leukocyte sample was reduced in all patients, with a mean activity of 1.22 ± 1.65 nmol/h/mg protein (range: 0.0-2.6 nmol/h/mg protein), consistent with severe enzyme deficiency.

### Molecular analysis

**Fig. 2:**
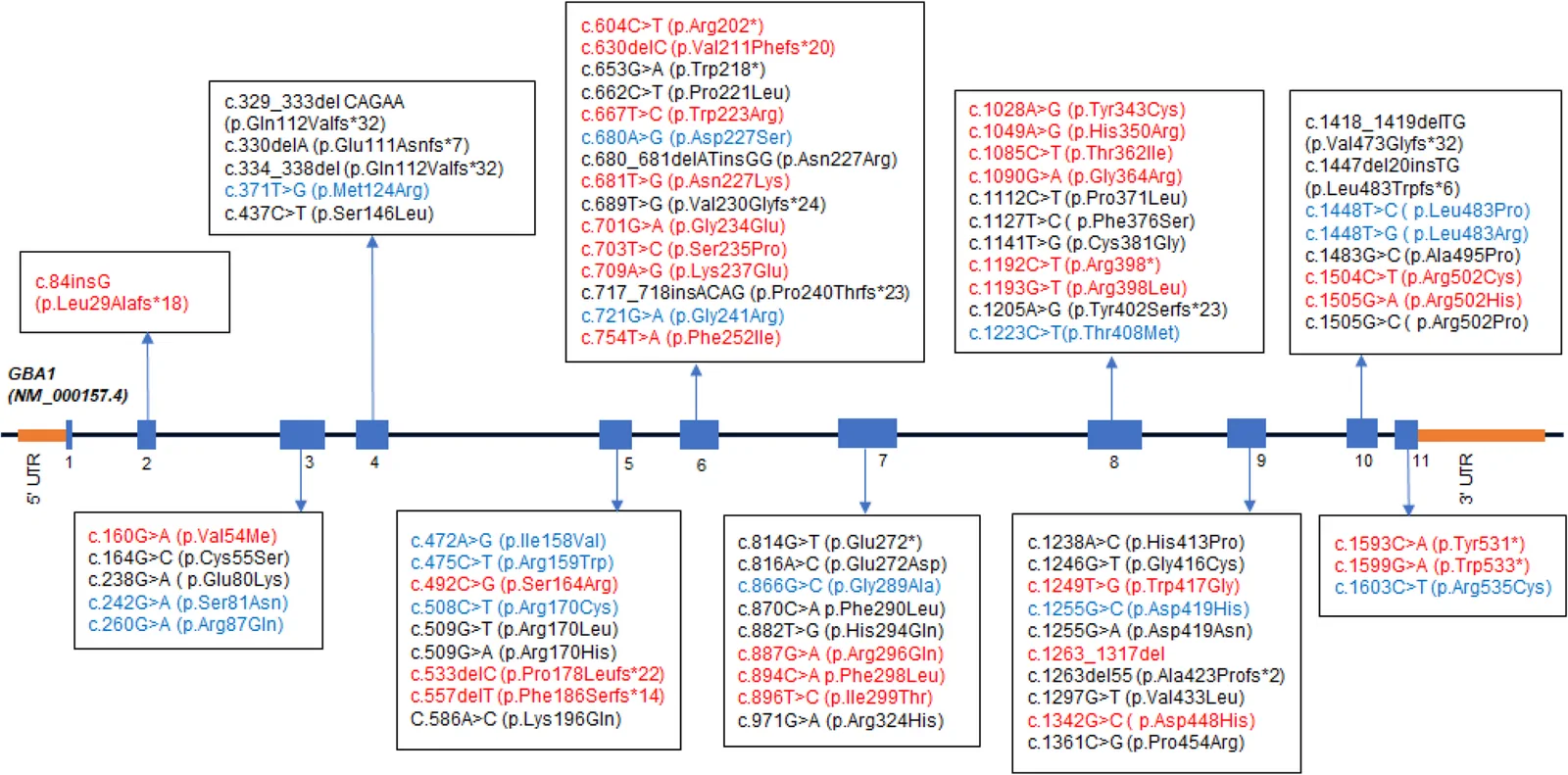
Schematic representation of the variants identified in the *GBA1* gene in patients with GD2 until date. Pathogenic variants are highlighted in red; variants identified in the present study are shown in blue

Molecular study identified compound heterozygous variants in the *GBA1* gene in 13 patients, while five patients harbored homozygous variants. In one case, only a single heterozygous variant was identified (P15). The most frequent variant identified in the present cohort was c.1448T > C (p.Leu483Pro), detected in 11 of 19 patients, in the homozygous state (*n* = 1) and in the compound heterozygous state (*n* = 10). Additional variants that were identified in the present study were c.242G > A (p.Ser81Asn), c.260G > A (p.Arg87Gln), c.371T > G (p.Met124Arg), c.472 A > G (p.Ile158Val), c.475 C > T (p.Arg159Trp), c.508 C > T (p.Arg170Cys), c.680 A > G (p.Asn227Ser), c.721G > A (p.Gly241Arg), c.866G > C (p.Gly289Ala), c.1255G > C (p.Asp419His), c.1448T > G (p.Leu483Arg) and c.1603 C > T (p.Arg535Cys). Interestingly, two recombinant alleles were also identified by Sanger sequencing: RecNcil comprising the point mutations c.1448T > C, c.1483G > C and c.1497G > C (*n* = 4), and Complex-C, comprising the point mutations c.475 C > T, c.667T > C, c.681T > A, c.689T > G, c.703T > C, c.721G > A, c.754T > A (*n* = 2). Overall, variants were distributed across exons 3, 4, 5, 6, 7, 9, 10, and 11 with exon 10 being the most common (*n* = 11/19). Figure 2 provides a schematic representation of all *GBA1* variants reported in patients with GD2 to date, including those identified in the present study.

Importantly, a rare recurrent missense variant, c.371T > G (p.Met124Arg) in exon 4, previously reported by our group [19] in two patients (P9 and P10), was identified in two additional unrelated patients (P12 and P13), both from Gujarat. The p.Met124Arg was further evaluated using multiple computational tools. In-silico analysis using MutPred2 assigned the p.Met124Arg variant a pathogenicity score of 0.872, with significant predicted effects including alteration at protein interfaces, membrane-associated processing sites, metal binding regions, and conserved motif architecture. The sequence conservation and alignment depth plot showed that this variant lies within the N-terminal conserved region, which is characterized by high sequence identity across species, consistent with a role in early protein folding, stability, and lysosomal targeting. Additionally, iStable tool predicted decreased protein stability (prediction score = 0.57), indicating a destabilizing structural effect consistent with impaired folding and reduced functional enzyme availability. Overall, the analysis indicated localized alterations in residue packing and interaction patterns. This pattern suggests that p.Met124Arg induces localized structural instability without causing major global conformational disruption of the GBA1 protein. Parental segregation analysis confirmed the variant to be present in *trans* with another pathogenic variant c.1448T > C in the *GBA1* gene. In addition, biochemical study showed reduced β-glucosidase enzyme activity in all four patients, providing supporting evidence of impaired *GBA1* function. Based on the ACMG-AMP classification system [25, 26] and ClinGen framework, the variant c.371T > G was classified as likely pathogenic based on the following criteria: PM1 (moderate), PM2 (supporting), PP3 (supporting), and PM3 (moderate). Although no direct in vitro functional assays specific to this variant have been performed, the combined genetic, biochemical, segregation, and computational evidence supports its classification as likely pathogenic.

## Discussion

Type 2 Gaucher disease (GD2) is a rare subtype characterized by early onset and rapidly progressive neurological involvement, with symptom onset before 6 months of age and death usually occurring by 2 years of age [14]. Data from the International Gaucher Registry (2000) reported that < 1% of patients had GD2 among 1,698 Gaucher patients [2], further highlighting the rarity of this disorder. Interestingly, a study from Turkey reported a relatively higher frequency of GD2 (4.9%) in their cohort [13]. Likewise, the proportion of GD2 was found to be 5.1% (19/375) of the total Gaucher patients diagnosed at our Centre over a period of 10 years. However, the observed proportion of 5.1% in the present study must be interpreted with caution. As our center serves as a specialized tertiary referral institution for lysosomal storage disorders, there is a high possibility of a referral bias toward more severe and acute phenotypes. Hence, this proportion may not accurately represent the true incidence of the GD2 subtype within the general Indian population, as many cases may still go undiagnosed. To the best of our knowledge, the present study represents the largest case series of GD2 patients reported from India to date (Additional file 4). Previously published Indian data have primarily focused on the broader Gaucher disease spectrum and there are only a few case reports of isolated GD2. Furthermore, this study provides insight into the phenotypic variability and expands the mutational spectrum of GD2 in Indian patients.

Using the classification system proposed by Donald et al. 2025, the patients in the present cohort were systematically categorized into the five groups namely Gaucher-related hydrops, neonatal inflammatory, neonatal neurodegenerative, early-infantile neurodegenerative, and late-infantile neurodegenerative GD. We identified the majority of cases as belonging to the early- and late- infantile neurodegenerative categories, which is similar to the trend observed in the recent review on 250 GD2 patients [10]. A subset of patients in the present cohort was classified as “possibly” early or late infantile neurodegenerative GD due to lack of clinical information, particularly the precise age of symptom onset. This classification is important to differentiate acute neuronopathic GD2 from GD3 phenotypes, especially in patients presenting beyond infancy or harboring GD3-associated variants as observed in patients P6, P16, P19. Nonetheless, the presence of progressive neurological deterioration, significant developmental impairment, and rapid disease progression supported their inclusion within the acute neuronopathic GD spectrum. Importantly, two cases of perinatal lethal GD2 were also documented in our cohort. According to the available literature, the perinatal form represents a relatively less frequently recognized but severe subtype, commonly associated with hydrops fetalis, collodion skin, hepatosplenomegaly, and early neonatal death. A previous study by Sheth et al. (2016) demonstrated the occurrence of lysosomal storage disorders in cases presenting with nonimmune hydrops fetalis, including one case of Gaucher disease [30]. However, the relatively small number of reported perinatal GD2 cases likely reflects underdiagnosis due to limited clinical awareness, short survival period, and overlap with other causes of hydrops fetalis.

The key presenting clinical features observed in GD2 patients in the present study included hepatosplenomegaly, thrombocytopenia, developmental delay or regression of milestones, oculomotor signs, and feeding difficulties, which are consistent with previously reported findings [11, 12, 14]. Splenomegaly is usually present along with neurological signs in GD2 and was observed in all patients in our cohort except those with the perinatal lethal form. However, a few GD2 cases reported in the literature presented initially with neurological signs in the absence of splenomegaly [9, 31]. This is an important clinical consideration; as such cases may be misdiagnosed. Conversely, the presence of isolated splenomegaly in infants should raise suspicion of GD2, as subtle neurological signs such as hyperextension of the neck or strabismus may be overlooked.

Most neurological manifestations in GD2 are attributed to brainstem degeneration caused by accumulation of toxic lipid metabolites, particularly glucosyl sphingosine within neurons and glial cells [32]. A notable limitation of the study is the lack of comprehensive neuroimaging data for all patients. While brain MRI findings were documented for one patient (P12), systematic neuroimaging studies were not performed across all cases due to the retrospective nature of the study. Future prospective studies incorporating longitudinal neuroimaging may provide further insights into the structural brain changes associated with the various clinical sub-categories of neuronopathic Gaucher disease. Feeding difficulties and strabismus are among the earliest and most prominent signs, whereas apnea typically occurs later in the disease course [9]. In our study too, feeding difficulties were observed as one of the common presenting signs. Apnea is a major cause of death and is associated with shorter survival when it occurs early in the disease course. This was evident in one patient in our cohort who developed apnea at later stage and had a family history of two siblings who died at 6 months of age, likely due to apnea.

Myoclonic seizures and failure to thrive are well-recognized features of GD2. Previous studies have suggested that myoclonus is more frequent in GD3 than in GD2, although its exact frequency in GD2 remains unclear [33, 34]. In our study, myoclonic seizures were observed in four patients. Failure to thrive was reported in 78% of GD2 patients in the series by Mignot et al. [9]. and it was also observed in all postnatal cases in the present cohort. Pulmonary involvement, primarily manifesting as chronic cough due to bronchorrhea and recurrent infections, has been reported in approximately 54% of GD2 patients [9]. Similarly, pulmonary manifestations were observed in three patients in the present study. Overall, the clinical outcomes observed in the present cohort reflect the fatal natural history of acute neuronopathic Gaucher disease, with 18 of 19 patients having died during the course of the disease. The one surviving patient received ERT for approximately four months without significant clinical improvement, leading to its discontinuation. This outcome is consistent with the well-established inability of currently available ERT agents to cross the blood–brain barrier, rendering them ineffective against the central nervous system pathology that drives morbidity and mortality in GD2 [35]. The age at death was not available for all deceased patients, which is a recognized limitation of the retrospective design of this study. Future prospective studies with systematic longitudinal follow-up would provide more complete survival data and better characterize the natural history of GD2 in the Indian population.

Bone marrow examination is often performed when Gaucher disease is suspected, as it is a relatively simple and safe method to identify Gaucher cells. However, Mignot et al. reported that Gaucher cells may not be detected in approximately one-third of GD2 cases, making glucocerebrosidase enzyme activity assay a more reliable diagnostic tool [9]. In the present study, bone marrow examination was performed in seven cases and Gaucher cells were identified in all. Two patients (P11 and P18) showed undetectable levels of plasma chitotriosidase activity. Previously, studies have shown a homozygous 24 bp duplication in the *CHIT1* gene is associated with absent chitotriosidase activity [36]. However, *CHIT1* gene sequencing was not carried out for any of the patients in the present study.

The molecular diagnostic approaches used in the present cohort included PCR-RFLP, Sanger sequencing, smMIP-based NGS, and WES study. The choice of methodology was guided by clinical presentation and availability of testing methods. Based on the earlier report by Sheth et al. 2014 and 2019 that showed the variant c.1448T > C (p.Leu483Pro) in ~ 60% of patients with GD type 1 in the Indian population [17, 19], PCR-RFLP was employed in all patients to first screen this common variant. In the present cohort, c.1448T > C (p.Leu483Pro) in exon 10 of the *GBA1* gene was also identified as the most common variant with an allele frequency of 31.5%. This variant is a well-established pathogenic variant and is recognized as a frequent pan-ethnic variant associated with severe disease [37, 19]. Subsequently, Sanger sequencing of the coding regions of the GBA1 gene was performed for further molecular characterization. Additional variants identified in the present study, including c.260G > A, c.475 C > T, c.508 C > T, c.721G > A, c.866G > C, c.1448T > G, and the RecNcil recombinant allele, have previously been reported to be associated with GD2 [10]. Although Sanger sequencing remains a widely used and reliable method for molecular diagnosis in clinical practice, it has several limitations. These include limited ability to detect large deletions, duplications, complex recombinant alleles and other structural rearrangements, particularly in genes such as *GBA1* that have a homologous pseudogene [38]. With the increasing adoption of NGS technologies in clinical diagnostics, targeted NGS panels, and WES are being increasingly utilized for the molecular diagnosis of genetically heterogeneous disorders. Two patients in the present cohort were diagnosed using these approaches. NGS-based methods provide high-throughput sequencing and enable simultaneous analysis of multiple genes, thereby improving diagnostic efficiency. However, these approaches also have limitations, including challenges in accurately detecting complex structural rearrangements, recombinant alleles, and copy number variations without the use of complementary methods or specialized bioinformatics pipelines [38]. These methodological limitations are particularly relevant in one perinatal GD2 case (P15- molecularly unresolved), where only a single heterozygous RecNcil allele was identified. It is possible that the second allele represents a complex recombinant or structural variant that could not be resolved by the molecular approach used at the time. Additional methods such as multiplex ligation dependent probe analysis (MLPA), long-range PCR, or long-read sequencing may improve detection of such variants and could be considered in future studies for comprehensive characterization of such unresolved cases [39].

Interestingly, the c.1255G > C (p.Asp419His) variant, previously reported in association with GD type 1 in Colombian patients [40], was identified in a GD2 patient in our cohort. In the Colombian patient, this variant was present in compound heterozygosity with p.Asn409Ser, which is typically associated with GD type 1 and a milder phenotype [41]. However, in the present study, we identified c.1255G > C in compound heterozygosity with c.1448T > C (p.Leu483Pro). It is possible that the more deleterious effect of the variant c.1448T > C contributed to the severe GD2 phenotype in this patient. Nevertheless, other genetic or environmental modifiers are likely contributors to phenotypic variability.

**Fig. 3:**
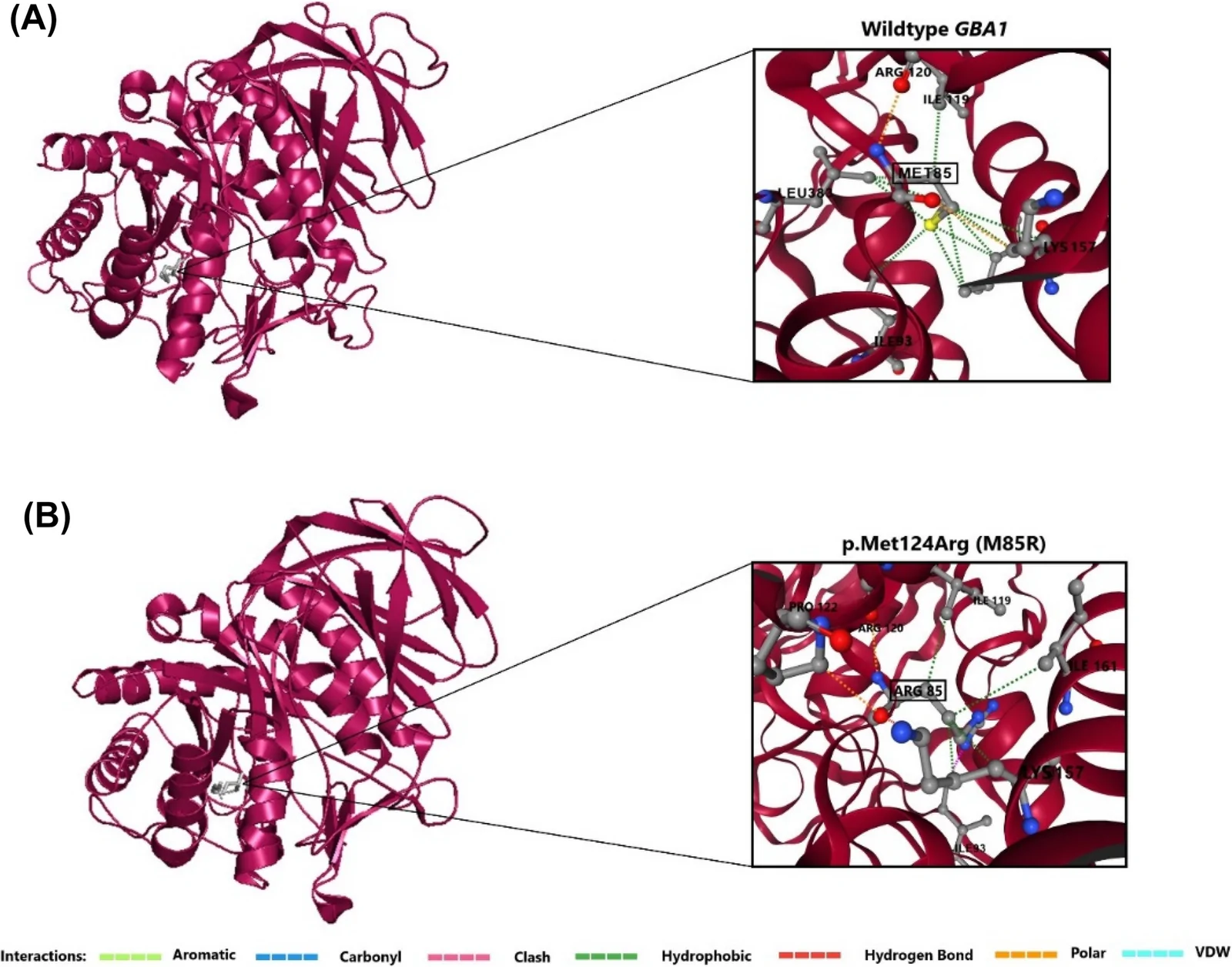
Structural and interaction analysis of GBA1 missense variant p.Met124Arg using PyMOL and DynaMut2. **A** Wild-type GBA1 protein depicting methionine at the native position (**B**) p.Met124Arg (M85R) mutant displaying modified local interaction networks. Left panels represent overall protein structure visualized using PyMOL. The target residues (wild-type and mutant) are highlighted in grey (stick representation), while the surrounding protein structure is shown as a magenta cartoon representation corresponding to Chain A of GBA1. Right panels represent local residue interaction networks predicted by DynaMut2. Interaction types are indicated as hydrogen bonds, hydrophobic interactions, polar contacts, aromatic contacts, Van der Waals interactions, carbonyl interactions, and steric clashes. Note: The target residue is labeled as M85R in the structural model to align with mature protein numbering, corresponding to p.Met124Arg in the text which follows the precursor protein numbering (including the 39-amino acid leader sequence)

An important finding in this study was the identification of a rare recurrent variant, c.371T > G, previously reported by our group [19] in two patients. In the present study, two additional unrelated patients from Gujarat were found to carry this variant in compound heterozygosity with c.1448T > C. This variant has not been reported in other GD cohorts to date, and in-silico analyses predict a deleterious effect. In-silico analysis predicted ΔΔG value of − 1.3 kcal/mol, which is suggestive of a moderate destabilizing effect on the protein structure. This may result in impaired folding efficiency, causing the protein to unfold and be degraded easily. Methionine resides within a compact hydrophobic core in the wildtype GBA1 protein. Multiple non-polar interactions between methionine and its neighboring amino acid residues have a stabilizing effect, promoting tight folding of the wildtype protein. Substitution of methionine with arginine in the hydrophobic core can result in decreased non-polar interactions. Arginine residue has a high propensity to introduce new polar interactions and hydrogen bonds, thus resulting in high steric strain. Overall, this substitution in the GBA1 protein resulted in shifts in local interactions, where hydrophobic interactions were replaced by polar contacts and hydrogen bonds, leading to a reconfigured packing arrangement while compromising local structural stability (Fig. 3). The regional clustering of this variant among putatively unrelated patients from Gujarat suggests a potential founder effect phenomenon. However, confirmation of this hypothesis by recruitment of a large regional cohort and haplotype analysis in future studies would provide evidence for the origin of the variant, such as that done for Morquio A and Tay-Sachs disease by our group previously [42, 43].

In two cases of perinatal lethal GD2 in our cohort, recombinant alleles in the *GBA1* gene were identified. This finding is consistent with previous reports, including a study by Chida et al., in which four patients with perinatal lethal GD carried homozygous recombinant alleles and died within 24 h of birth [44]. These observations support a strong association between the presence of recombinant alleles in the *GBA1* gene and the perinatal lethal form of GD2. Thus, despite a small cohort size, a notable genotype-phenotype correlation was observed. Particularly, presence of homozygous recombinant alleles is associated with the severe, perinatal lethal form of GD, as seen in P14 in the present study. This is consistent with published reports documenting homozygous recombinant alleles (e.g., RecNcil/RecNcil) in association with the hydropic phenotype [44]. In contrast, 10 patients in our study who carried the p.Leu483Pro variant in compound heterozygosity with another missense variant or complex allele showed a later clinical presentation. These patients were classified either into the early-infantile or late-infantile neurodegenerative groups as per the classification proposed by Donald et al. 2025. This finding is also consistent with that observed in the literature that identified the p.Leu483Pro/ RecNcil as the most frequent genotype observed in early-infantile neurodegenerative GD. In addition to this, p.Leu483Pro / c.115 + 1G > A, p.Leu483Pro / p.Gly241Arg and RecNcil/ RecNcil are the other recurrent genotypes seen in patients with GD2. Importantly, p.Leu483Pro remains the consistent variant on one allele in the majority cases, which is similar to the observation in the present study. This suggests that while p.Leu483Pro is a marker for severe neuronopathic disease, the identity of the variant on the second allele influences the clinical course and degree of severity. Additional file 2 describes the key clinical features observed in cases with these recurrent genotypes. Although a definite genotype-phenotype correlation is difficult to establish, the observations highlight key clinical pointers associated with these recurrent genotypes in GD2. Of note, variants previously associated with GD type 1 (c.1255G > C) and GD type 3 (c.721G > A, c.680 A > G) were observed in GD2 patients in our cohort. These findings highlight the significant phenotypic variability in Gaucher disease and suggest that disease severity cannot be predicted solely based on genotype, and may be influenced by additional modifying factors such as noncoding SNPs within the *GBA1* gene that regulate protein expression in peripheral tissues [45].

An important association between Gaucher disease and Parkinson disease (PD) has been demonstrated by recent studies, identifying *GBA1* as one of the genetic risk factors for PD [46]. Both individuals with biallelic pathogenic variants in the *GBA1* gene causing GD as well as heterozygous carriers have been observed at an increased risk of developing PD compared to the general population [47]. However, the estimated risk varies across studies and despite the increased relative risk, the majority of individuals carrying monoallelic or biallelic *GBA1* variants might not develop Parkinsonism during their lifetime [47, 48]. The present study comprised patients with pathogenic variants in the *GBA1* gene leading to GD2 phenotype. However, detailed longitudinal clinical information regarding adult-onset neurodegenerative disorders, including PD, was not systematically collected for extended family members. None of the families reported a confirmed history of PD at the time of evaluation. Nevertheless, the parents of these patients are heterozygous carriers of *GBA1* variants and may have an elevated risk for developing PD later in life. Awareness of this association, combined with long-term neurological follow-up and genetic counselling, could be beneficial for affected families.

## Conclusion

This study represents the largest case series of type 2 Gaucher disease reported from India to date and provides important insights into its clinical and molecular characteristics in the Indian population. The findings highlight the early and severe clinical presentation of GD2, with hepatosplenomegaly, failure to thrive, and progressive neurological involvement being the most consistent features. Molecular analysis demonstrated a predominance of the c.1448T > C (p.Leu483Pro) variant, frequently observed in compound heterozygosity with other pathogenic variants, reaffirming its strong association with severe phenotypes. The identification of a recurrent c.371T > G (p.Met124Arg) variant in unrelated patients from Gujarat suggests the possibility of a founder effect and expands the mutational spectrum of GD2 in India. Additionally, the presence of recombinant alleles in cases with perinatal lethal presentation further supports their association with the most severe form of the disease. Overall, the observed clinical and genetic heterogeneity underscores the complexity of genotype-phenotype correlations in GD2 and highlights the importance of integrating clinical evaluation with enzymatic and molecular testing for accurate diagnosis. Further studies, including haplotype analysis and larger multicentric cohorts, will be valuable in confirming potential founder variants and improving understanding of disease variability in this population.

## Supplementary Information

Additional File 1: List of primers used for PCR-RFLP for the common variant c.1448T>C (p.Leu483Pro) in the GBA1 gene and primers used for Sanger sequencing of exons 1-11 of the GBA1 gene.

Additional File 2: Key clinical signs observed in patients with GD2 harboring recurrent genotypes in the GBA1 gene.

Additional File 3: ClinVar Accession ID of the variants identified in the given study. The variants identified through Sanger sequencing are reported in NCBI ClinVar database. The file provides accession ID and the links to the individual variant. 

Additional File 4: Summary of the previously published GD2 studies in different cohorts.

Additional File 5.

## Data Availability

The dataset generated and/or analyzed during the current study is available in the ClinVar repository (See Additional file 3).

## Abbreviations

GD: Gaucher disease
GD2: Type 2 Gaucher disease
smMIP: Single molecule molecular inversion probe
NGS: Next generation sequencing
DNA: Deoxyribonucleic acid
WES: Whole exome sequencing
