# Supplementary material for: Clinical, biochemical and molecular spectrum of acute neuronopathic type 2 Gaucher disease from India
Source: BMC Pediatr. 2026 Jun 27;26:884. doi: 10.1186/s12887-026-07232-4 (PMC13587318; doi:10.1186/s12887-026-07232-4)
Supplement: Supplementary file 1 [file 12887_2026_7232_MOESM3_ESM.docx]

The dataset generated and/or analyzed during the current study is available in the NCBI ClinVar repository. Following are the accession ID of the variants submitted to the ClinVar repository

1. **Variant c.1603C>T (p.Arg535Cys) in exon 11 of *GBA1* gene.**

ClinVar Accession ID: SCV000281966.1

[<https://www.ncbi.nlm.nih.gov/clinvar/variation/242383/>]

2. **Variant c.1448T>G (p.Leu483Arg) in exon 10 of *GBA1* gene.**

ClinVar Accession ID: SCV000786646.1

[<https://www.ncbi.nlm.nih.gov/clinvar/variation/93449/>]

3. **Variant c.721G>A (p.Gly241Arg) in exon 6 of *GBA1* gene**

ClinVar Accession ID: SCV007540275

<https://www.ncbi.nlm.nih.gov/clinvar/variation/93459/>

4. **Variant c.472A>G (p.Ile158Val) in exon 5 of *GBA1* gene**

ClinVar Accession ID: SCV007540276

5. **Variant c.260G>A (p.Arg87Gln) in exon 3 of *GBA1* gene**

ClinVar Accession ID: SCV004801944.2

<https://www.ncbi.nlm.nih.gov/clinvar/variation/2503944/>

6. **Variant c.475C>T (p.Arg159Trp) in exon 5 of *GBA1* gene**

ClinVar Accession ID: SCV007540277

7. **Variant c.242G>A (p.Ser81Asn) in exon 3 of *GBA1* gene**

ClinVar Accession ID: SCV007540278

8. **Variant c.371T>G (p.Met124Arg) in exon 4 of *GBA1* gene**

ClinVar Accession ID: SCV007540279

9. **Variant c.1255G>C (p.Asp419His) in exon 9 of *GBA1* gene**

ClinVar Accession ID: SCV007540280

<https://www.ncbi.nlm.nih.gov/clinvar/variation/4817706/>

10. **Variant c.866G>C (p.Gly289Ala) in exon 7 of *GBA1* gene**

ClinVar Accession ID: SCV000282512.4

<https://www.ncbi.nlm.nih.gov/clinvar/variation/236406/>

11. **Variant c.508C>T (p.Arg170Cys) in exon 5 of *GBA1* gene**

ClinVar Accession ID: SCV007540281

<https://www.ncbi.nlm.nih.gov/clinvar/variation/93453/>

12. **Variant c.680A>G (p.Asp227Ser) in exon 6 of *GBA1* gene**

ClinVar Accession ID: SCV007540282

<https://www.ncbi.nlm.nih.gov/clinvar/variation/4314/>

13. **Variant c.1448T>C (p.Leu483Pro) in exon 10 of *GBA1* gene**

ClinVar Accession ID: SCV000786647.2

<https://www.ncbi.nlm.nih.gov/clinvar/variation/4288/>
